# TALEN-Mediated FLAG-Tagging of Endogenous Histone Methyltransferase DOT1L

**DOI:** 10.4236/abb.2017.89023

**Published:** 2017-09-22

**Authors:** Cheng An, Guangjing Zhu, Suzanne N. Martos, Xue Feng, Haimou Zhang, Yankai Jia, Zhibin Wang

**Affiliations:** 1 Guang’an Men Hospital, China Academy of Chinese Medical Sciences, Beijing, China; 2 Laboratory of Human Environmental Epigenomes, Department of Environmental Health Sciences, Bloomberg School of Public Health, Johns Hopkins University, Baltimore, MD, USA; 3 School of Life Sciences, Hubei University, Wuhan, China; 4 GENEWIZ Suzhou, Suzhou, China; 5 Fenxian Central Hospital, Shanghai, China; 6 Department of Oncology and Sidney Kimmel Comprehensive Cancer Center, School of Medicine, Johns Hopkins University, Baltimore, MD, USA

**Keywords:** DOT1L, Flag, TALEN, Knock-In

## Abstract

Histone modification including H3 lysine 79 methylation (H3K79me) plays a key role during gene transcription and DNA damage repair. DOT1L, the sole methyltransferase for three states of H3K79me, is implicated in leukemia, co-lorectal cancer, and dilated cardiomyopathy. However, understanding of DOT1L and H3K79me in these pathways and disease pathogenesis has been limited due to the difficulty of working with DOT1L protein. For instance, locus-specific or genome-wide binding sites of DOT1L revealed by chromatin immunoprecipitation (ChIP)-based methods are necessary for inferring its functions, but high-quality ChIP-grade antibodies are currently not available. Herein we have developed a knock-in approach to tag endogenous DOT1L with 3 × Flag at its C-terminal domain to follow functional analyses. The knock-in was facilitated by using TALENs to induce a targeted double-strand break at the endogenous DOTIL to stimulate local homologous recombination at that site. The single cell colonies with successful knock-in were isolated and verified by different methods. We also demonstrated that tagged DOT1L maintains its normal function in terms of methylation and that the engineered cells would be very useful for further studies.

## 1. Introduction

### 1.1. DOT1L and Its Functional Roles

Histone lysine methylations play a key role in gene transcription [1] [2] [3] and they are catalyzed by a group of histone lysine methyltransferases (KMTs). Yeast Dot1 (disruptor of telomeric silencing; or Kmt4) [4] or its mammalian homolog DOT1-Like (DOT1L) is the sole KMT in their respective genome with methylation activities toward H3K79 inside globular domain of histones. Both enzymes can catalyze mono-(me1), di-(me2), and trimethylation (me3) in a non-processive manner [5] [6]. In addition to histone methylation, DOT1L can methylate other protein factors for regulation. For example, methylation of androgen receptor at lysine 349 is involved in the regulation of androgen receptor responsive genes [7] [8]. DOT1L plays pivotal roles in embryonic development, hematopoiesis, and cardiac function [9]. Due to its role in development, it is not surprising to find that DOT1L is linked to many human diseases, including prostate cancer, colorectal cancer, and hypertension [7] [8] [10] [11] [12]. Interestingly, high level of DOT1L expression and H3K79me2 was a predictor of poor patient survival of colorectal cancer[11].

Considering the importance of DOT1L, the genome-wide location analysis (using ChIP-Seq) of DOT1L will provide detailed information to infer its function. However, the lack of specific, ChIP-Seq grade antibodies of DOT1L (Drs. Yi Zhang and T-p Chen, personal communications [13]) hinders such genome-wide analysis. To solve this lack of high quality antibody, one approach is to over express Flag- or Myc-tagged DOT1L and then use anti-Flag or Myc for ChIP assays. For example, we have used anti-Flag to map the location of DACH1, a protein with roles in breast cancers, in breast cancer cell line MDA- MB-231 with stably expressed Flag-DACH1 [14]. However, this over expression approach is limited with difficulty in maintaining a physiologically relevant expression level. Flag- or Myc-tagging the endogenous gene would avoid the aforementioned limitation. In addition, the small size of Flag sequences seem to seldom affect the target protein, and the availability of high affinity anti-Flag antibodies make the ChIP assays highly successful. Furthermore, endogenous tagging will reveal the true function of the protein compared to over expression studies[15] [16].

Current methods of knock-in are limited by low homologous recombination (HR) efficiency even when adeno-associated virus (AAV) was used as a vector to provide the donor plasmid for HR. Apart from the complexity of virus packaging, isolation of hundreds of colonies followed by verification by PCR is laborious and may result in only a few successful knock-in colonies. A targeted double strand break (DSB) has reported to increase HR efficiency by 2000 ~ 10,000 fold [16]. Herein, we induced a DSB using TALEN (described below) to facilitate tagging of endogenous DOT1L with 3 × Flag sequences to generate single colony cell lines that express DOT1L-3 × Flag endogenously.

### 1.2. TALEN-Mediated Genome Editing

Since the original reports on the creation and use of zinc finger nucleases (ZFNs) to stimulate locus-specific genome editing by Chandrasegaran group [17] [18], we have seen dramatic progress in the field of genome engineering. It is laborious and time-consuming to generate highly specific ZFNs for desired target sites. The fortuitous discovery of transcriptional activator-like effectors (TALEs) [19] [20] has expanded and simplified the generation of custom TALE DNA- binding domains with programmable specificity [21] [22] [23]. Repeat monomers in TALE with amino acids at position 12 and 13 bind to a specific nucleotide (e.g., NI to A, HD to C, NG to T, and NN to G or A). The TALE motifs are then linked in tandem to generate custom TALE binding domains that target a specific sequence. These are then coupled to Fok I nuclease domain (to form TALENs) or other effector domains (such as transcription factors: TALE-TF) providing a versatile platform for achieving a wide variety of targeted genome manipulations specificity that include gene knock-out, knock-in, activate and repress gene expression, respectively [21] [22] [23] [24] [25]. A pair of TA-LENs separated by 14 – 20 bp are designed to induce a targeted DSB at the desired chromosomal locus, which will be repaired by one of the two cellular mechanisms: non-homologous end joining (NHEJ) or HR (when provided with an exogenous donor plasmid containing homologous sequences flanking the cut site).

Here, we report efficient tagging of the endogenous DOT1L with 3 × Flag using TALENs and a donor plasmid containing homologous sequences. We show successful knock-in of DOT1L-3 × Flag into HEK293 cells, which was verified by using different methods. However, low expression of endogenous DOT1L in HEK293 cells, limited the ChIP-Seq experiments for detecting genome-wide binding sites.

## 2. Materials and Methods

### 2.1. Construction of Donor Plasmid for Homologous Recombination

We followed a modified protocol from the method in reports [15] [16]. A ~1.1 kb genomic fragment upstream and 1.2 kb downstream of the stop codon of human DOT1L (transcript 006 of Ensembl release 69—October 2012, ENST00000440640) fragment was PCR amplified using platinum Taq polymerase High Fidelity (Invitrogen, USA) to form the left and right homologous arms, respectively. Refer to Table 1 for the information on primers. These were cloned into pAAV-USER-Neo-LoxP-3 × Flag vector (kind gift from Dr. Zhenghe Wang at Case Western Reserve University) using the USER system (NEW ENGLAND BioLabs, USA) as previously described elsewhere [15] [16]. This recombinant plasmid served as the DOT1L donor plasmid without packaging into AAV for the knock-in study. The donor plasmid was sequenced to verify the sequences of the left and right homology arms.

### 2.2. Construction of TALEN Pairs Targeting the Stop Codon Region of DOT1L

TALEN pairs targeting the stop codon regions of DOT1L were constructed based on the system developed in a previous report [22]. The plasmids for TALEN system was ordered from Addgene as denoted in the protocol [19]. We designed one pair of TALEN targeting the following sequence of the human DOT1L gene: 5-TATGCCGCGCACCTTTCGG gggttaagccgcgataa AGACCTTGCTTAGCTAGCA-3 where the uppercase nucleotides show the binding sites for TALEN left (TN-1L, 5 end) and right (TN-1R, 3 end) arms, respectively; and the lowercase letters represent the spacer sequence between the left and right arms of the TALEN pair. The stop codon (taa) is underlined. We also designed an additional TALEN pair (TN-2L and TN-2R) targeting 5-TGCCGCGCACCTTTCGGGGgttaagccgcgataaagaccttGCTT AGCTAGCAGTGCGTA-3. The combination of TN-1L and TN-2R generates a third TALEN pair for targeting. The genes encoding the TALE motifs are shown in Table 2.

### 2.3. Cell Culture and Transfection

HEK293 cells (ATCC^®^ CRL-1573^™^, US) were cultured in Dulbecco’s Modified Eagle Medium containing 10% FBS and 1% penicillin and streptomycin (Life Technologies, USA), and maintained in a humidified incubator (5% CO_2_) at 37°C.

For the knock-in (KI) study, HEK293 cells were transfected with 2 μg of TALEN left arm and right arm plasmids using GenJet^™^ In Vitro DNA Tranfection Reagent (Ver. II, SiganaGen^®^ Laboratories). After transfection, the cells were cultured for 48 hours to extract genomic DNA to monitor the cutting efficiency of the TALEN pairs (TN-1L/1R; TN-2L/2R; TN-1L/TN-2R). For the knock-in study, HEK293 cells were transfected with 2 μg of TN2L and TN2R plasmids, and 2 μg donor plasmids using GenJet^™^ In Vitro DNA Transfection Reagent.

### 2.4. Verification of TALEN Cutting Efficiency Using the Surveyor Nuclease

As reported previously[22], genomic DNA of TALEN pairs-transfected HEK293 cells were PCR amplified around the stop codon region with primer pair F2 and R2. The resulting PCR product contains a heterogeneous population (649 bp) of both TALEN-modified and unmodified DNA. Heteroduplexes of both amplified DNA were generated after denaturing and then re-annealing slowly. Surveyor nuclease (from Transgenomic, USA) cleaves the heteroduplexes at TALEN targeting sites (stop codon region) with mismatches to produce two fragments (~219 and 430 bp in size), while leaving homoduplexes intact. The two fragments were separated by gel electrophoresis and visualized under UV.

### 2.5. Monoclonal Knockin Cell Selection and Verification

After transfection with donor plasmid and TALEN plasmids, cells were selected with Geneticin 418 disulfate salt (G418, Singma-Aldrich, USA) for 48 hours, and then the cells were dispersed and serially-diluted in 96-well plates with medium containing G418 for single colony selection as reported elsewhere [15] [16]. The G418 resistant single cell clones were then screened for HR by PCR using the primer pairs F1/NR and NF/R1 for the left and right arms, respectively. Refer to Table 1 for the sequences of the primer pairs. Only cells that originated from a single cell colony containing the correct knock-in sequence were used in later study.

### 2.6. Western Blot

For Western blot, whole cell lysate of knock-in cell lines were extracted as described elsewhere [26]. 30 μg or 150 μg total protein extract was loaded on SDS-PAGE gel (BIO-RAD, USA) to separate the proteins according to their sizes. The proteins from the gel were then transferred to a PVDF membranes and incubated with primary antibodies of Flag (1:1000, F3165, Sigma), DOT1L (1:4000, A300-953A, Bethyl), H3K79me2 (1:1000, Ab3594, Abcam), H3K79me3 (1:1000, Ab2621, Abcam) and *β*-actin (1:5000, A5441, Sigma) at 4 C overnight. After washing with 1 × TBST (BIO-RAD, USA), the membranes were incubated with horseradish peroxidase-labeled secondary antibodies (GE healthcare, UK and EPITOMICS, USA) and then detected with ECL western blotting detection reagent (GE healthcare, UK).

## 3. Results

### 3.1. Overview of the Knock-In Strategy

**Figure 1:**
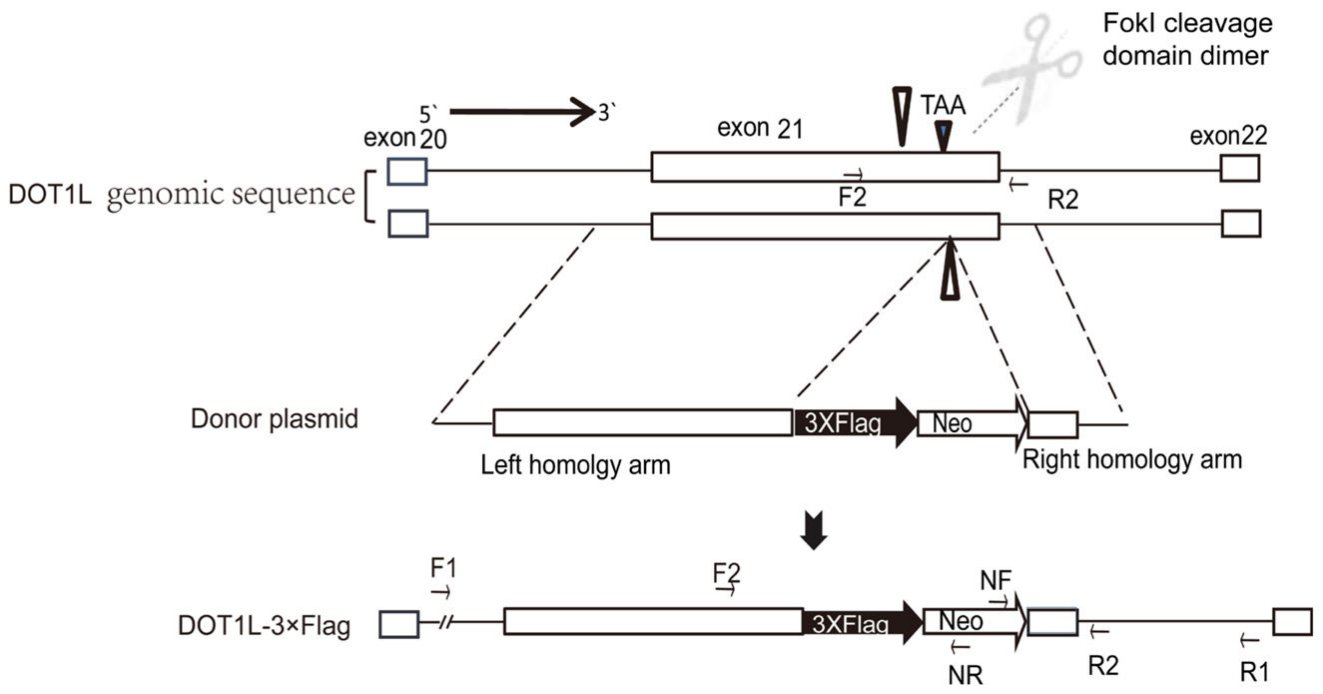
Schematic diagram of knockin strategy. The strategy involved 3 steps: first, TALEN pairs were used to create DSB at the stop codon region of DOT1L to facilitate HR. The left and right arm of TALEN pairs recognize regions before and after the stop codon, and dimerized FokI endonuclease generates a site specific double strand break to facilitate DOT1L-3 × Flag gene knock-in through HR. Second, donor plasmid contains a left and right arm homologous to sequences in human DOT1L, flanking a Neo-Lox P-3 × Flag cassette was constructed. Donor plasmid was transfected along with the TALEN pair plasmids to induce a targeted DSB for HR. Third, successful knock-in single colony was picked and verified by PCR. Note that drawing is for viewing purpose only and it is not proportional to the size of genomic sequences.

Our objective was to tag C-terminal end of endogenous DOT1L with 3× Flag sequences and use anti-Flag antibody to pull down endogenous DOT1L for future analyses of DOT1L function in HEK293 cells. To achieve this, we first cloned ~1.1 kb sequences upstream and ~1.2 kb sequences downstream of the stop codon (including the stop codon) of DOT1L from HEK293 genomic DNA into pAAV-USER-Neo-LoxP-3 × Flag vector using the USER system to use as a donor plasmid (see Figure 1) [15] [16]. The cloned donor plasmid contains 3 × Flag sequences just before the stop codon and neomycin resistance genes for selection of positive knock-in clones with G418 treatment. We used TALENs to induce a targeted DSB and transfected the donor plasmid into the cells to stimulate HR. As expected, the DSB was readily repaired by HR in presence of the donor plasmid; we observed highly efficient HR occurs at the DSB. Knock-in cells were further serially diluted and selected by G418 treatment. The correct knock-in single colonies were identified by PCR and sequencing. The strategy for the knock-in is shown in Figure 1.

### 3.2. TALEN Pair Design, Construction and Verification

**Figure 2:**
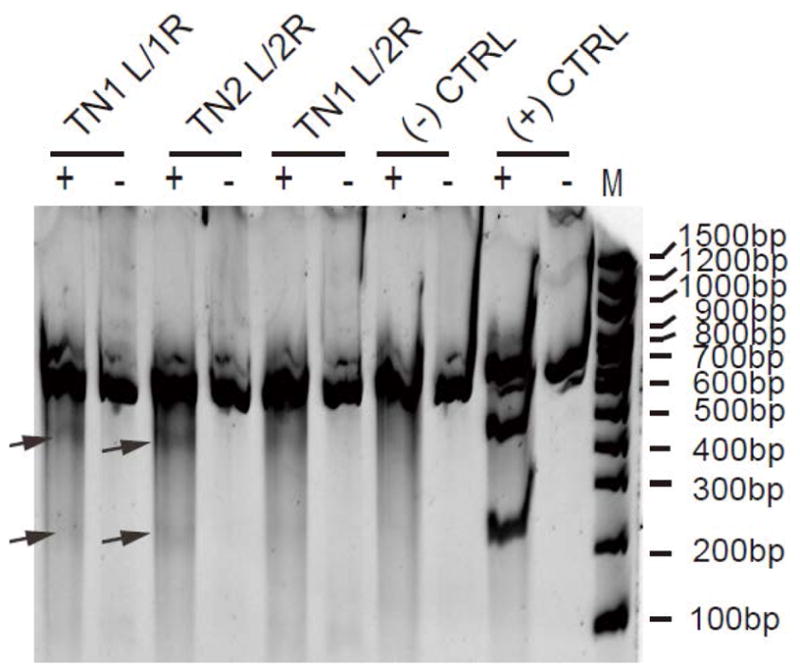
Surveyor nuclease assay to verify the DSB induced by TALEN pairs. Genomic DNA of HEK293 cells transfected with TALEN pair plasmids were PCR amplified around the stop codon regions using primer pair F2 and R2 as shown in Figure 1. Surveyor nuclease treatment of successfully KO cells produces 2 segments (~219 bp & 430 bp, arrows). “+” and “−” represent with or without surveyor nuclease treatment, respectively. Negative and positive controls in the kit were verified the same way, with or without surveyor nuclease treatment. Surveyor nuclease digestion products were 217 bp and 416 bp for the positive control.

Traditional knock-in methods rely on natural HR; however, the efficiency is very low even when using AAV as a vector for delivery of the donor plasmid. It has been reported that a targeted DSB could increase HR efficiency by 2000 ~ 10,000 fold [16]. We used the TALENs to create targeted DSB in the genome of HEK293 cells. We induced a TALEN-mediated targeted DSB at the stop codon region of DOT1L (Figure 1) within the genome of HEK293 cell line and provided the requisite donor plasmid for HR (incorporating a 3 × Flag sequence after the stop codon of DOT1L gene). For knock-in strategy, we designed three pairs of TALENs (TN1L/1R, TN2L/2R, and TN1L/2R) targeting the stop codon region and verified their cleavage efficiency by surveyor nuclease assay (see Figure 2). All three TALEN pairs were shown to create a DSB, but with different efficiency: TN2L/2R pair was the most efficient among the three pairs and it was used for the knock-in study.

### 3.3. TALEN-Mediated Knock-In of 3 × Flag Sequences to the Endogenous DOT1L

**Figure 3:**
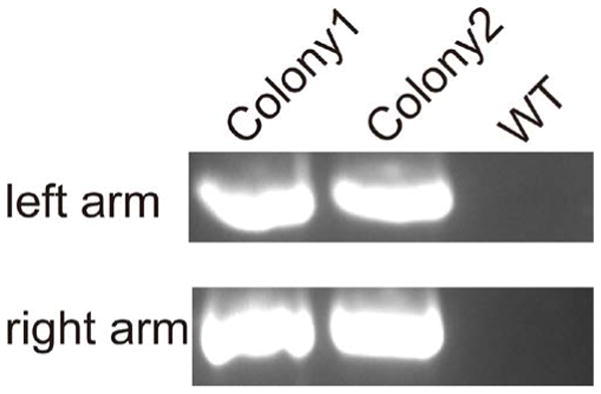
PCR verification of successful knock-in single colony cell lines. Genomic DNAs of single colony cells after knock-in were used to screen with primer pairs F1/NR and NF/R1, producing expected PCR products of 1712 bp and 1750 bp, respectively, confirming the correct HR at the DOT1L site.

**Figure 4:**
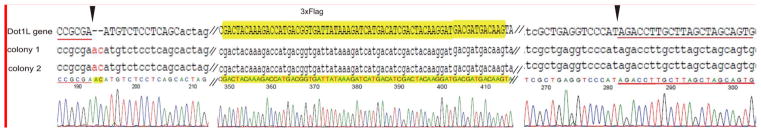
Sequencing results from successfully KI cell. Left panel shows the junction of right flank of endogenous and left flank of plasmid and “ac” were added to guarantee fusion gene in frame. Red underline represents the sequence of Dot1L-P1R. Middle panel shows the sequence of 3 × Flag knocked in. Right panel shows the junction of left flank of endogenous and right flank of plasmid. Redunderlin represents the sequence of Dot1L-P2F.

We transfected HEK293 cells with this TALEN pair (TN2L/2R) in presence of a donor plasmid (containing 3 × Flag sequences before the stop codon of endogenous human DOT1L) for HR. Single colonies were selected with G418 selection. Two representative colonies were selected for further study. Two pairs of PCR primers (F1 and NR for left arm, NF and R1 for right arm) were used to verify the correct insertion of donor plasmid sequences within the genome (see Figure 1 & Figure 3). Only successful knock-in cell showed the right sized PCR products for both arms; the knock-in sequences of genomic were verified by Sanger sequencing (see Figure 4).

### 3.4. Verification of DOT1L-3 × Flag Expression after Knock-In

**Figure 5:**
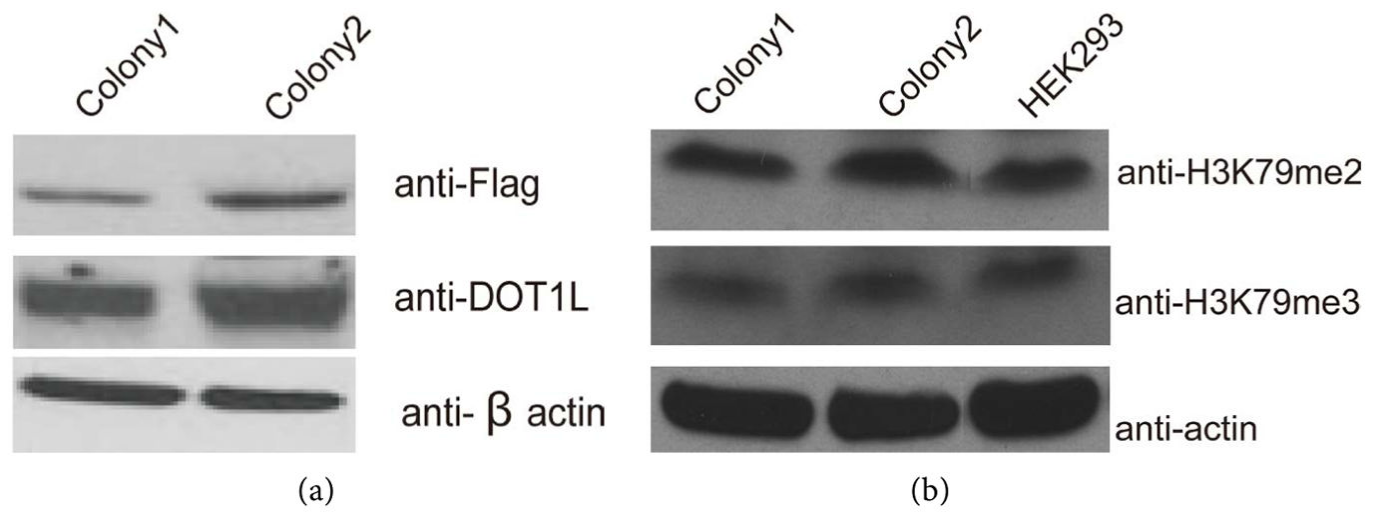
Function verification of DOT1L-3 × Flag knock-in cell lines (a). Whole cell lysate of single colony cell lines expressing DOT1L-3 × Flag were used for Western blot using anti-Flag antibodies (150 μg whole cell lysate). *β*-actin was used as a control (bottom). (b). Western blot of H3K79me2 and H3K79me3 were done using whole cell lysate of DOT1L-3 × Flag knockin cell lines and the knock-in did not change the methylation status of H3K79me2 and H3K79me3.

To verify the successful expression of DOT1L-3 × Flag, we did Western blot analysis with anti-Flag antibody using two representative knock-in single colonies. We were unable to detect DOT1L signal when we used 30 μg of total protein (a routine loading amount in our experiments) from whole cell lysate. We reasoned that the failed detection might be due to low expression level of DOT1L-3 × Flag. DOT1L-3 × Flag protein was then enriched by immunoprecipitation of whole cell lysates of knock-in cell lines with anti-Flag antibody. The Flag-enriched whole cell lysate was monitored by anti-DOT1L antibody; we successfully detected the DOT1L protein of the correct size in the knock-in cell lines, but not the control HEK293 cells (data not shown). Therefore, we reason that the expression of DOT1L-3 × Flag after knock-in is relatively low. When we increased the quantity of total proteins loaded into the gel to 150 μg, we could successfully detect the signal of DOT1L-3 × Flag using anti-Flag antibody cell lysates, without the need for enrichment (see Figure 5(a)).

### 3.5. Functional Verification: DOT1L-3 × Flag Knockin did not Change H3K79me2 and H3K79me3 Levels

DOT1L is the only enzyme that is responsible for (mono-, di- and tri-) methylation of H3K79. To verify that DOT1L-3 × Flag knock-in did not change the normal function of DOT1L, we extracted whole cell lysate of knock-in cell lines and examined H3K79me2 and H3K79me3 levels by Western blot. We found that knock-in did not change the expression of both methylation states of H3K79 (see Figure 5(b)), suggesting that DOT1L-3 × Flag functions similarly to endogenous DOT1L after KI.

## 4. Discussion

### 4.1. Pros and Cons of Knock-In

Although most systematic studies of mammalian proteins relied on ectopic expression of target proteins, these expressed proteins may lack important regulatory sequences such as 3′ UTR or introns that are driven by constitutive or inducible promoters. Thus, they are quite different from the endogenous status and might provide results that are artifacts due to overexpression or non-endogenous regulation of tagged proteins. Epitope tagging of endogenous proteins circumvents this problem and has shown its power and convenience in different research areas such as epigenetics [12] [13] and proteomics [18]. However, the challenge is the relatively low targeting rates (2% ~ 5%) even when AAV virus was used for HR in cells [15] [16].

Endogenous epitope tagging provides an accurate platform for exploring various functions of proteins. However, most proteins have multiple transcripts which could be translated into proteins of different sizes that might function diversely. But, it is not uncommon that alternative transcripts share the same stop codon in the genome, which is tagged by 3 × Flag as in our case. Thus, one could tag a group of transcripts sharing the same stop codon. It is a fact that, to fully explore the profile of each transcript and its corresponding protein, laborious work needs to be done to tag different transcript groups with different stop codons. In this case, according to the Ensembl release 69 when the project was initiated, DOT1L has 9 splicing variants with 6 protein coding transcripts and among them, transcripts DOT1L-006, -008 and -201 share the same stop codon, which was targeted for HR in our study. However, for the same gene, it is not always a good idea to choose the stop codon shared by most transcripts since they are not necessarily similarly abundant in mRNA level or functionaly importantly at the protein level. RNA-Seq or microarray data would be helpful when it is available for the target genes. Our results here only could be explained by the 3 transcripts mentioned above and the lower expression of these 3 transcripts may be one reason for the failed ChIP-Seq experiment. Our ability to detect peaks could be limited by insufficient reads to detect a true difference in this model. Low endogenous expression of DOT1L combined with the non-specific binding of anti-Flag antibody could explain our inability to identify peaks.

### 4.2. Genomic Editing Techniques

The discovery of Zinc-finger nuclease [17] ushered in the era of genome engineering also known as genome editing, which was expanded further by TALENs [22]. The development of CRISPR/Cas system has made genome editing much simpler and easier [23]. In this study, we improved the previous knock-in technique [15] [16] by using TALENs, which creates double strand breakages (DSBs) of the targeted gene and was shown to increase the recombination efficiency significantly in many genomic regions [26]. We successfully tagged DOT1L with 3 × Flag with the USER system and further verified its proper function in this study. However, the lower expression of endogenous DOT1L in HEK293 cells limited our ChIP-Seq experiments with anti-Flag. A different cell line with highly expressed DOT1L will be a better model for future studies.

### 4.3. Key Steps for This Strategy

High efficiency knock-in is ascribed to the targeted DOT1L DSB using the TA-LENs. We suggest several improvements for efficient knock-in. First, the high sequence similarity between TALE motifs hinders rapid construction of the TALEN pairs. We purified TALE hexamers away from monomers after PCR reaction using gel extraction and PCR product purification kits. TALENs generated using these TALE hexamers almost always yield the correct genes, which has been confirmed by sequencing. Second, seed cells are no more than 60% confluence before transfection using TALEN pair and donor plasmid. Our suggestion is direct conversion of normal medium to that containing 1 mg/ml G418 after 48 hours. It is important that avoid treatment with trypsin. Third, pick colony with tip to resuspend in medium containing 1 mg/ml G418 and then serially dilute in a 96 well plate. After 12 hours, we need to monitor the wells containing single cell colonies under an inverted microscope to guarantee the positive cells arose from a single cell colony.

## 5. Conclusion

The strategy for knock-in based on the TALEN technique we showed is a high efficiency and successful. It is facilitated to induce a targeted double-strand break at the endogenous DOTIL to stimulate local homologous recombination at that site. The single cell colony with tagged DOT1L maintains its normal function in terms of methylation and that the engineered cells would be very useful for further studies.

## Figures and Tables

**Table 1:** Primers sequence for knock in and verifying test.

Primer Name	Sequences	Size (bp)	Uses
DOT1L-P1R	GGA GAC A/ideoxyU/GTTCGCGGCTTAACCCCCGAAA	1079	Left arm
DOT1L-P1F	GGG AAA G/ideoxyU/TGCACGGTGGCGAACTCCAG		
DOT1L-P2R	GGC ATA G/ideoxyU/TCCTAAGAGACCCAGCATAG	1152	Right arm
DOT1L-P2F	GGTCCCA/ideoxyU/AGACCTTGCTTAGCTAGCAG		
F1	CAGGTTCCCTTCCGCACTCT	1713	picking positive colony
NR	GTTGTGCCCAGTCATAGCCG		
NF	TCTGGATTCATCGACTGTGG	1750	picking positive colony
R1	TAGTTACCTGCAGAAGGGCA		
F2	CCGCCTGCTAACGCCTCTTT	649	Surveyor nuclease cutting
R2	ACGCCACCCGTCATGAGTGA		

**Table 2:** TALEN pairs information.

TN pairs	Arms/Sequences	Sequences
TN1	Left	NI NG NH HD HD NH HD NH HD NI HD HD NG NGNG HD NH NH
	Right	NH HD NG NI NH HD NG NI NI NH HD NI NI NH NH NG HD NG
	Sequence	T ATGCCGCGCACCTTTCGG gggttaagccgcgataa AGACCTTGCTTAGCTAGC A
TN2	Left	NH HD HD NH HD NH HD NI HD HD NG NGNG HD NH NHNHNH
	Right	NI HD NH HD NI HD NG NH HD NG NI NH HD NG NI NI NH HD
	Sequence	T GCCGCGCACCTTTCGGGG gttaagccgcgataaagacctt GCTTAGCTAGCAGTGCGT A
